# The national economic burden of rare disease in the United States in 2019

**DOI:** 10.1186/s13023-022-02299-5

**Published:** 2022-04-12

**Authors:** Grace Yang, Inna Cintina, Anne Pariser, Elisabeth Oehrlein, Jamie Sullivan, Annie Kennedy

**Affiliations:** 1 The Lewin Group, 3160 Fairview Park Drive, Suite 600, Falls Church, VA 22042 USA; 2 National Center for Advancing Translational Sciences, National Institutes of Health, DHHS, 6701 Democracy Boulevard, Suite 206, Bethesda, MD 20892 USA; 3 National Health Council, 1730 M St NW, Suite 500, Washington, DC 20036 USA; 4 EveryLife Foundation for Rare Diseases, 1012 14th NW, Suite 500, Washington, DC 20005 USA

**Keywords:** Rare disease, Economic burden, Direct cost, Indirect cost

## Abstract

**Background:**

To provide a comprehensive assessment of the total economic burden of rare diseases (RD) in the United States (U.S.) in 2019. We followed a prevalence-based approach that combined the prevalence of 379 RDs with the per-person direct medical and indirect costs, to derive the national economic burden by patient age and type of RD. To estimate the prevalence and the direct medical cost of RD, we used claims data from three sources: Medicare 5% Standard Analytical File, Transformed Medicaid Statistical Information System, and Optum claims data for the privately insured. To estimate indirect and non-medical cost components, we worked with the rare disease community to design and implement a primary survey.

**Results:**

There were an estimated 15.5 million U.S. children (N = 1,322,886) and adults (N = 14,222,299) with any of the 379 RDs in 2019 with a total economic burden of $997 billion, including a direct medical cost of $449 billion (45%), $437 billion (44%) in indirect costs, $73 billion in non-medical costs (7%), and $38 billion (4%) in healthcare costs not covered by insurance. The top drivers for excess medical costs associated with RD are hospital inpatient care and prescription medication; the top indirect cost categories are labor market productivity losses due to absenteeism, presenteeism, and early retirement.

**Conclusions:**

Our findings highlight the scale of the RD economic burden and call for immediate attention from the scientific communities, policy leaders, and other key stakeholders such as health care providers and employers, to think innovatively and collectively, to identify new ways to help improve the care, management, and treatment of these often-devastating diseases.

**Supplementary Information:**

The online version contains supplementary material available at 10.1186/s13023-022-02299-5.

## Background

It is estimated that there are more than 7000 rare diseases (RDs) affecting about 30 million Americans [1]. While the exact cause for many RDs remains unknown, for a large number of RDs the origin can be traced to mutations in a single gene, contributions from multiple genetic factors, and/or a combination of genetic and environmental factors [2]. Besides the direct medical costs associated with RD, there are significant costs to society and individuals, including indirect costs associated with productivity losses, non-medical costs such as spending on home or motor vehicle modifications, and certain healthcare costs not covered by insurance. Many individuals with RDs have high medical needs requiring that they miss work, retire early, and utilize the assistance of a caregiver for activities for daily living [3, 4]. Caregivers also experience work productivity losses to fulfill their caregiving responsibilities [5]. As such, the economic burden of RD is likely to be significant, for patients, unpaid family caregivers, and society.

There is limited evidence on RD prevalence and economic burden in the United States (U.S.) because of a lack of a national registry or all-payer database enabling the calculation of disease prevalence or cost. Although data sources such as the Europe-based Orphanet^1^ contain prevalence estimates for many RDs, these data may or may not apply to the U.S. population and do not always contain details such as the age-specific prevalence. A literature review also found that most RD-related cost studies are Europe-based and often focus on a small number of high-cost RDs [6–8]. Cost of illness information guides healthcare decision-makers in quantifying the impact of diseases at a population level. This can inform resource allocation, as well as healthcare cost projections [9]. However, data on the burden of RDs in the U.S. is scarce.

To address this knowledge gap, we estimated the economic burden of RD in the U.S. in 2019 for a group of 379 RDs identified via the National Economic Burden of Rare Disease Survey (hereafter, the Survey). While the existing literature typically focus on individual RDs, we took a comprehensive approach by estimating the economic burden of these RDs in aggregate. Historically, due to the small prevalence of individual RDs, and the heterogeneity in disease etiology, clinical presentation, healthcare needs, and cost implications across difference RDs, rare diseases were viewed as diseases that may be individually debilitating but with limited public health implications. Advances in science and research have now shown several commonalities across RDs: first, many RDs share the same genetic or environmental risk factors [2]; second, there is evidence that therapeutic approaches that work for multiple RDs are both feasible and cost-effective [10]; third, patients with different RDs often share similar clinical experiences and care journeys. For instance, the phenomenon often regarded as the extensive “diagnostic odyssey” is known to affect many RD communities and results in significant delay in appropriate treatment, emotional stress, and economic loss [11]. Such new evidence supports the argument that rare diseases are not rare. A comprehensive view of the economic burden of RDs as a group will help public health policy leaders and other key decision-makers understand the magnitude and impact of RD, inform the direction of required resource allocation, and provide key insights as to short- and long-term policy opportunities and solutions for the RD community. Such collective data may also serve to motivate other stakeholders such as drug developers to find synergy in research and development of new treatments. Therefore, this study aims to provide the most comprehensive estimate of the total economic burden of RD as a group and from a societal perspective, in addition to expanding the evidence base in those less well-understood cost components and caregiver burden.

## Results

An estimated 15.5 million individuals in the U.S. have at least one of the 379 RDs that were included in this study in 2019. The estimated RD prevalence of 379 RDs is reported in Table 1.

**Table 1:** Rare disease prevalence for 379 RDs included in the study by age and insurance coverage, in 2019

	No. of persons estimated to have RD	Population	Prevalence (%)
Age			
< 18	1,322,886	71,580,109	1.8
18–64	8,371,639	182,528,781	4.6
≥ 65	5,850,660	51,822,242	11.3
Insurance			
Commercial	7,124,610	188,738,510	3.8
Medicaid	1,582,062	57,833,466	2.7
Medicare	6,838,513	59,359,156	11.5
Total	15,545,185	305,931,132	5.1

*Source*: Analyses of 2018 de-identified Normative Health Information(dNHI) claims, a large claims database for the privately insured, 2019 Medicare claims, 2016 Medicaid claims, and Census population projection for 2019 (the latest available at the time of this study)

**Fig. 1:**
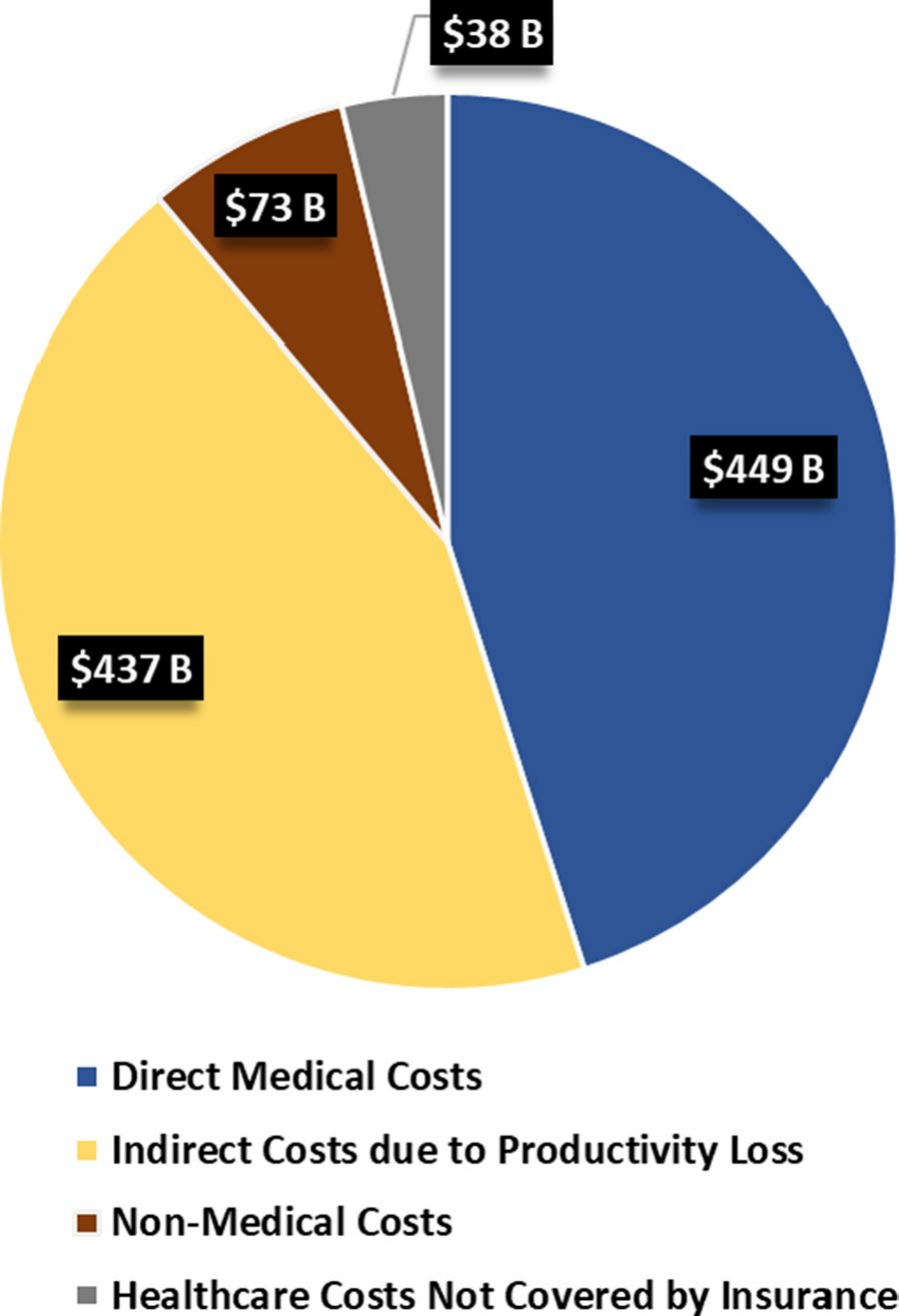
Total Economic Burden of Rare Disease in the U.S. in 2019: $997 Billion

The estimated total economic burden of 379 RDs in 2019 was $997 billion, including a direct medical cost of $449 billion and an additional $548 billion in indirect, non-medical costs, and healthcare costs not covered by insurance (non-covered costs). Figure 1 shows the estimated total economic burden of RD by cost components. The direct medical cost was calculated as the excess medical costs due to RD, from comparing patients with RD and their matched controls without RD and comprised of total medical and prescription drug costs from administrative claims. Indirect costs due to productivity losses for both patients and family caregivers included earnings losses due to early retirement, absenteeism (days missed from work), presenteeism (days affected by disease and felt unproductive), social productivity loss in volunteer work, and education costs (i.e., homeschooling and special education, for pediatric patient sample only). Non-medical costs included costs such as home or vehicle modifications. Lastly, non-covered costs included costs such as costs of acupuncture, massage therapy, medical foods, and dietary supplements that the insurance do not cover.

The direct medical cost of RD represents almost half of the total burden (45%), followed by indirect costs due to productivity loss (44%), non-medical costs (7%), and healthcare costs not covered by insurance (4%).

### Direct medical costs

Table 2 shows that the working-age population with commercial health coverage bears majority of the direct (excess) medical cost of RD (47%). The Medicare population with RD accounts for about 43% of total direct costs, and the Medicaid population with RD accounts for the remaining 10%. The estimated $449 billion for direct medical cost translates into a per-person excess cost of $28,913, implying that an average person with RD has an annual medical cost that is $28,913 more than the comparison group without RD. The per-person excess medical cost of RD decreases with age, with children on average having an annual excess cost of $32,037 when they have RD; $29,647 for working-age adults with RD, and $27,157 for RD patients age 65 and older. Across different insurance coverages, the highest per-person excess costs are for the privately insured ($29,910), followed by cost for the Medicare beneficiary population with RD ($28,185); the lowest per-person excess costs are for persons with RD who are on Medicaid ($27,573).

**Table 2:** Direct medical cost of rare diseases by age, insurance coverage, and type of service, in 2019

	Total excess medical cost due to RD		Per-person (2019 $)
	(in Million $)	Percentage of the total (%)	
Age			
< 18	42,381	9.4	32,037
18–64	248,198	55.2	29,647
≥ 65	158,884	35.3	27,157
Insurance			
Medicaid	43,621	9.7	27,573
Commercial	213,094	47.4	29,910
Medicare	192,747	42.9	28,185
Type of service			
Inpatient	143,000	31.8	9199
Prescription medication	79,466	17.7	5112
Outpatient	62,032	13.8	3990
Other ancillary	48,974	10.9	3150
Outpatient prescription administration	47,567	10.6	3060
Physician	31,372	7.0	2018
Non-acute inpatient	30,759	6.8	1979
Durable medical equipment	4,401	1.0	283
Caregiver	1890	0.4	122
Overall	449,462	100	28,913

*Source*: RD prevalence calculated from claims data:2018 de-identified Normative Health Information (dNHI) claims, a large claims database for the privately insured, 2019 Medicare 5%, and 2016 Medicaid, combined with the 2019 Census population projections; direct medical cost estimates also based on three claims databases. Other ancillary services include telehealth, ambulance transportation via land, air or water, mobile unit services, etc.

Hospital inpatient care and prescription medication are the two largest cost categories, representing 32% and 18% of the total direct medical cost, respectively. Durable medical equipment (DME) and caregiver costs represent the two smallest categories. Caregiver costs, for hired aid, were only covered by Medicaid; however, we estimated them as an average value for the entire RD population, to be consistent with other cost categories.

### Indirect costs due to productivity loss

RD may increase the likelihood that severe functional impairment or disability will prevent people with RDs from working, limit employment opportunities, and reduce earnings [3, 4]. We designed and implemented the National Economic Burden of Rare Disease Survey (i.e., the Survey) to collect data on productivity loss, non-medical costs, and healthcare costs not covered by insurance. Our survey results indicated that among the working-age (18–64) persons with RD, 43.8% are in the labor market, as compared to the national labor force participation rate of 63.1% among the U.S. adult population.

As shown in Table 3, the estimated total indirect cost of RD is $437 billion in 2019, with $46 billion to children with RD, and $391 billion to adults with RD, both including the patients’ own productivity loss and that of their unpaid family caregivers. Absenteeism for both persons with RD and their caregivers is nearly $149 billion (34% of the $437 billion), followed by presenteeism cost ($138 billion, 32%), and losses due to early retirement ($136 billion, 31%). For adults, the costs of absenteeism for caregivers are about the same as those for the person with RD ($64 billion versus $60 billion). However, when the costs to caregivers for children with RD are accounted, the costs of absenteeism for the caregivers surpass those for the person with RD ($89 billion versus $60 billion). Losses associated with presenteeism are slightly smaller for caregivers compared to the person with RD ($63 billion across all caregivers for children and adults with RD versus $75 billion for persons with RD). Due to the significant costs in absenteeism, presenteeism, and social productivity loss to family caregivers of children with RD, the per-person indirect cost is higher for children with RD than for adults with RD ($34,448 vs. $27,501).

**Table 3:** Total and per-person indirect costs by cost component, in 2019

	Age < 18			Age ≥ 18		
	Person with a RD	Primary Caregiver	Secondary Caregiver	Person with a RD	Primary Caregiver	Secondary Caregiver
Indirect cost due to productivity loss						
Total (in Million $)			45,571			391,126
Early retirement	NA	850	1083	88,877	38,462	6823
Absenteeism	NA	10,265	14,497	59,853	50,176	14,024
Presenteeism	NA	10,176	7232	74,741	40,453	5367
Social productivity loss in volunteer work	494	550	424	8226	4035	89
Per-person (2019 $)			34,448			27,501
Early retirement	NA	642	818	6249	2704	480
Absenteeism	NA	7759	10,959	4208	3528	986
Presenteeism	NA	7692	5467	5255	2844	377
Social productivity loss in volunteer work	373	416	321	578	284	6

Source: RD prevalence calculated from claims data: 2018 de-identified Normative Health Information (dNHI) claims, a large claims database for the privately insured, 2019 Medicare 5%, and 2016 Medicaid, combined with the Census population projection for 2019; indirect and non-medical costs estimated from the Survey data

### Non-medical costs and healthcare costs not covered by insurance

Table 4 shows that the total non-medical cost is $73 billion and healthcare costs not covered by insurance is $38 billion. Among the non-medical costs, special equipment at home or on a personal/family vehicle (e.g., wheelchair) represented the largest share (32%), followed by transportation cost (28%), necessary home modification (e.g., ramp, 14%), and paid daily care (12%). Healthcare costs not covered by insurance included family spending on things such as experimental, alternative, or non-traditional treatments; over-the-counter drugs; or dental surgeries.

**Table 4:** Total and per-person non-medical costs and healthcare costs not covered by insurance, in 2019

	Total (in Million $)		Per-Person (2019 $)	
	Age < 18	Age ≥ 18	Age < 18	Age ≥ 18
Non-medical costs	16,285	56,990	12,310	4007
Paid daily care (i.e., paid assistance with daily living)	1482	7477	1121	526
Necessary home modification (e.g., ramp, stair lifts)	1682	8709	1271	612
Special equipment at home or on a personal/family vehicle (e.g., wheelchair, shower chair, hydraulic commode lift)	1865	21,677	1409	1524
Transportation costs (e.g., costs incurred while seeking care or attending clinical trials)	1305	19,127	986	1345
Home schooling (i.e., expenses related to home schooling if the child with RD cannot attend normal school due to RD)	454	NA	344	NA
Missed schooling (i.e., days missed from preschool or school in an average school month because of rare disease)	2298	NA	1737	NA
Special education (e.g., school-based special services to the affected child such as speech and language therapy, braille books, or sign language interpreter)	7199	NA	5442	NA
Healthcare Costs Not Covered by Insurance (e.g., family spending on experimental, alternative, or non-traditional treatments; over-the-counter drugs; or dental surgeries)	2172	35,750	1642	2514

*Source*: RD prevalence calculated from claims data (2018 dNHI, 2019 Medicare 5%, and 2016 Medicaid) combined with the Census population projection for 2019. Non-medical costs^a^ and healthcare costs not covered by insurance were estimated from the Survey data

^a^Non-Medical Cost components include: (1) Missed school: Days missed from preschool or school in an average school month because of rare disease. (2) Home schooling: expenses related to home schooling if the affected person cannot attend school or paying for a nanny beyond what would have been spent if not because of the rare disease. (3) Transportation costs: Increased transportation costs (e.g., driving to and from clinics or specialized facilities, attending clinical trials, traveling to patient community meetings, medical conferences, or advocacy events, parking, etc.). (4) Home modification: Expenses on home modifications (e.g., barrier free lift systems, stair lifts, automatic door openers, ramps, technology to enable access through an X-box or iPad, adaptations for hearing or vision impairments, other). (5) Special equipment at home: Expenses related to purchasing/installing/modifying special equipment at home or on a personal family vehicle (e.g., bathroom equipment such as a shower chair, commode chair, hydraulic commode lift, modification to the wheelchair such as elevated leg rests, modified joysticks and switches, automated/raised desk trays, vehicle modifications to accommodate driver or passenger with disability, etc.). (6) DME: Expenses related to purchasing equipment (e.g., pulse oximeter, suction machine, habilitation equipment such as standers, alternative pressure air mattress, motorized hospital bed, etc.). (7) Special education: services that school provide special care to the affected person, either via informal supports, or via a 504 Plan or an IEP. The special education services include: Full-time or part-time personal care attendant for the classroom, occupational or physical therapy, speech and language therapy, special education supports and auxiliary aid, equipment such as augmentative communication or technology supports, etc.

The average non-medical cost is $12,310 for children with RD and $4007 for adults with RD. Average healthcare costs not covered by insurance is $1642 per child with RD and $2,514 per adult with RD.

Additional file 1 shows that for patients age 18 or younger, the total per-person cost ranges from $71,921 for “Diseases of the musculoskeletal system and connective tissue” to $189,010 for “Lysosomal storage diseases”. For adult patients age 18 and older, the total per-person cost ranges from $40,844 for “Diseases of the eye and adnexa” to $92,065 for “Lysosomal storage diseases”. Direct medical cost has a much larger variation across RD groups than per-person indirect cost, non-medical costs, or healthcare costs not covered by insurance.

## Discussion

We showed that the total economic burden of 379 RDs approaches $1 trillion in 2019, representing a substantial impact on the U.S. economy, from a societal perspective. Data demonstrated that RD imposes significant costs to individual households and the health system in the forms of excess medical cost, productivity loss to both individuals and unpaid caregivers, and expenses in mitigating challenges of daily life, e.g., home modification for easier access. Our findings can help guide healthcare decision-makers in setting up and prioritizing healthcare policies and interventions related to RDs.

Our estimate of the overall economic burden is based on a subset of 379 RDs and, therefore, represents a lower bound estimate and is not generalizable to RDs not included in this study. The previous burden estimates are generally limited to a specific RD or a small group of RDs and U.S.-based cost estimates are very scarce. Excluding cystic fibrosis and hemophilia, which are relatively well studied, information on the economic cost for other conditions (e.g., Duchenne muscular dystrophy, fragile X syndrome, juvenile idiopathic arthritis, mucopolysaccharidosis, scleroderma, Prader-Willi syndrome, histiocytosis, epidermolysis bullosa) is very limited [12].

Previous studies that target a specific disease produce cost estimates with very wide ranges. For example, Kawalec and Malinowski’s (2015) systematic review of the indirect costs related to psoriatic arthritis, showed that per person indirect cost range was $1694–$12,318 using the friction cost approach and $1751–$50,270 (in 2013 $s) using the human capital approach [13]. In our study, psoriatic arthritis is included in the “Diseases of the skin and subcutaneous tissue” group, with estimated indirect costs of $22,066 per adult with such conditions. We were not able to identify U.S.-based studies that estimated the overall burden of RD, but a few studies focused on the burden of specific diseases. For example, aggregate inpatient costs among eleven genetic orphan diseases in the U.S. were roughly $1 billion in 2016, with cystic fibrosis and sickle cell disease having the largest costs ($414.8 million and $338.2 million) [14].

Most people living with RDs in this study were between the ages of 18 and 65. While commercial payers bore the largest share of the direct medical cost, indirect and non-medical costs paid by families were greater than direct costs. Significant productivity losses associated with absenteeism ($149 billion) and presenteeism ($138 billion) by patients and family caregivers alike were experienced by employers. Non-medical costs ($73 billion) such as spending on paid daily care, necessary home modification (e.g., ramp), special equipment at home or on a personal/family vehicle (e.g., wheelchair), or special education (for children only) represent the largest burden on the families with a loved one affected by RD, because these costs are typically paid out of pocket. Healthcare costs not covered by insurance ($38 billion), such as costs of non-traditional treatments, although modest compared to other cost components, could add to the financial pressure of the families affected. Finally, though excluded from the estimated total burden, we also estimated the costs to government supplemental income programs that provide disability support to persons with RD whose ability to participate in the labor market is affected by RD. This total cost is estimated to be roughly $115 billion.

Our study has several limitations. Firstly, RD prevalence and direct medical cost estimates relied on one instance of a diagnosis code, which may include false-positive cases (i.e., persons with a singular diagnosis code who were later ruled out as having a RD). While we captured as many RDs as possible, this may have resulted in an overestimate of the prevalence, and/or an underestimate of the direct medical costs (as patients with one diagnosis code may not be as severe as those with multiple diagnosis codes of RD in a given year).

Secondly, not every RD was matched to a definitive International Classification of Disease 10^th^ Edition (ICD-10) code. Some RDs we matched to the closest ICD-10 code, which might have captured a broader group of people than intended. Conversely, in some instances the absence of a specific ICD-10 code for a RD required mapping to a disease category that was not rare, thus eliminating it from inclusion in the direct cost analysis. The lack of specific ICD code for the majority of rare diseases often adds to the overall rare disease burden by impacting the availability of disease data to inform payer decision making, diagnostic and treatment algorithms, and public health surveillance.

Thirdly, we used the prescription drug costs of the 60–64-year-olds with private insurance to impute the average drug costs for Medicare eligible individuals 65 or older. This will likely underestimate the true medication costs for older patients with RD.

Additionally, the indirect and non-medical costs were based on the self-reported survey data. Since it is a convenience sample, there may be selection biases if people who are more severely affected by a RD are more likely to respond to the survey than those with less severe RD which creates an overestimation bias. Conversely, if only the experience of individuals with confirmed RD diagnoses and the ability to participate in a rigorous online, self-report survey are represented in the survey sample, there could be an underestimation bias.

It should be noted that this study focuses on the RD burden in 2019 and does not reflect the cumulative costs associated with living with a RD over time.

Finally, due to small sample sizes, we could not break down burden estimates by desired population strata (e.g., sex, race/ethnicity).

Despite these limitations, this study is the largest and most comprehensive effort so far in the U.S. to measure the societal impact of many RDs at once, encompassing various cost components (such as cost of social productivity loss and cost of special education), and including estimates of indirect productivity loss for family caregivers whose lives are significantly affected by RD.

## Conclusions

Together, the 379 RDs included in this study represent an economic burden that far surpasses some of the costliest chronic diseases studied in the U.S., both due to a high prevalence (i.e., more than 15 million individuals with these rare diseases) and a higher per-person cost. The findings of this study highlight the scale of the RD burden and call for immediate attention from the scientific communities, policy leaders, and other key stakeholders such as health care providers and employers, to think innovatively and collectively, and to identify new ways to help improve the care and treatment of RD. These findings demonstrate that the RD community has a significant unmet need with tremendous public health impact. This need is one that requires urgent support to advance the research and development of resources for the prevention of comorbidities, management, and ultimately, cures of these often-devastating diseases. All these steps may lead to significant individual and societal benefits.

## Methods

**Fig. 2:**
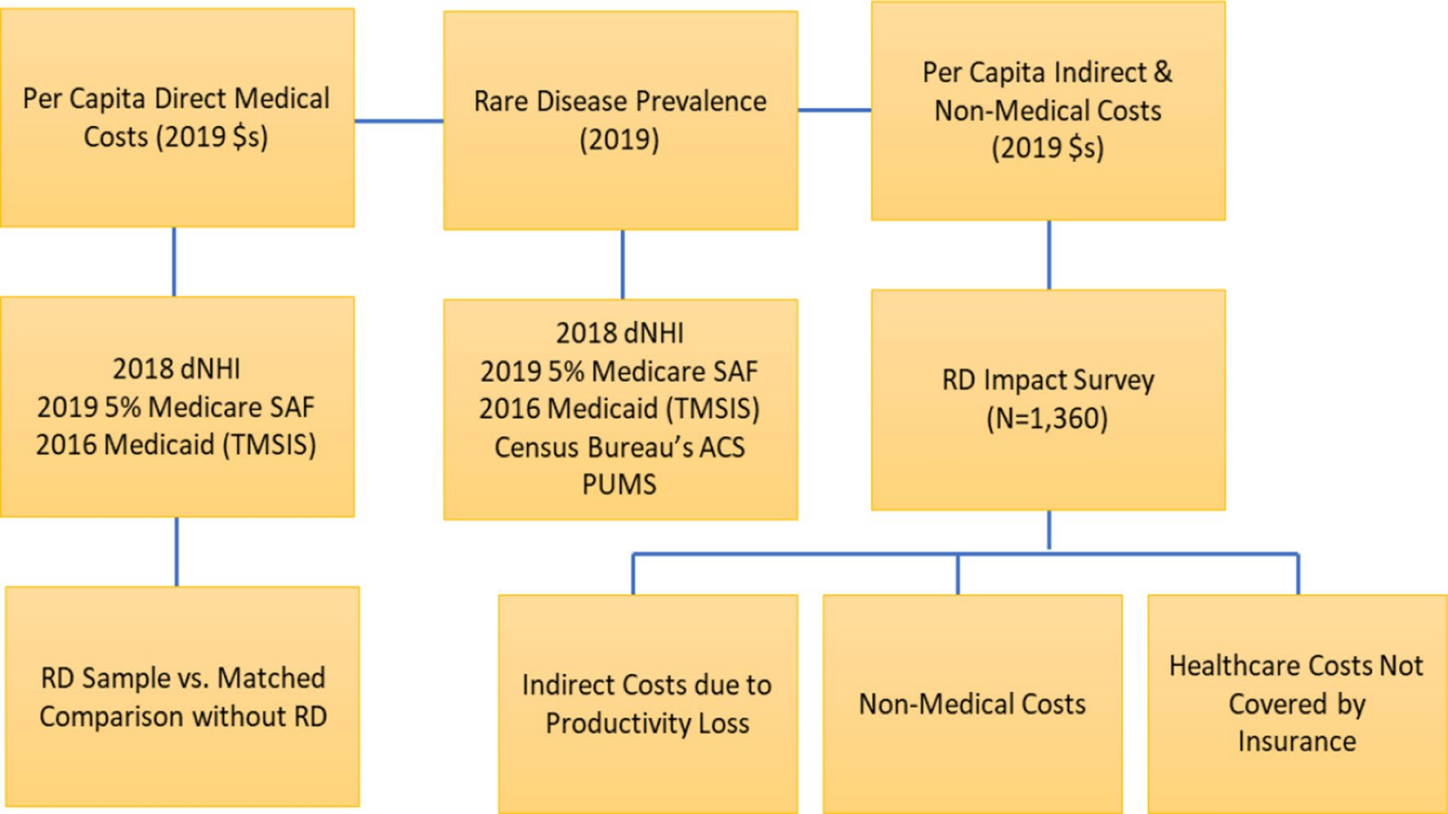
Data sources for the economic burden estimates

To estimate the national economic burden by patient age and type of RD, we took a prevalence-based approach. This approach combined the RD prevalence with per-person disease-attributable excess cost (i.e., a difference in the annual per-person costs between the RD sample and matched controls without RD). Direct medical costs were captured through an administrative claims-based analysis. The indirect productivity losses for persons with RD and their caregivers, non-medical costs, and healthcare services costs not covered by insurance were captured via the survey that we designed and implemented– the National Economic Burden of Rare Disease Survey. This survey was one of the largest surveys conducted so far covering multiple communities representing 379 RDs. The study received approval from an Institutional Review Board. Figure 2 shows the data sources used to identify the cost categories.

We developed a process to assign ICD-10 codes to each individual RD and then map them to RD groups. We first consolidated the disease names, synonyms, and acronyms for the 400 + individual RDs reported by the Survey respondents, and then compared the RD names against existing disease ontologies. We relied on clinical experts’ opinion to determine the appropriate name for disease terms that could not be matched to existing disease ontology and excluded those that were anomalies, e.g., entry errors, disease is not an RD, and names of phenotype or genes that may be associated with multiple diseases. These resulted in a “clean” list of 379 RDs. We then mapped each disease to its corresponding ICD-10 code. We then ran the ICD-10 code designation against the unique clinical coding system for RDs maintained by the Orphanet (see footnote 1) to identify ICD-10 codes for diseases that remained unassigned, and to validate the entire list against Orphanet code designation. Lastly, we mapped the final 379 RDs to 16 disease groups (see Additional file 2) representing a body system based on the ICD-10 diagnosis coding system. The entire process went through several rounds of review by a designated technical advisor group consisting of clinical experts, RD researchers, and family advocates.

### RD prevalence

Because there is not a single dataset that addresses the prevalence of multiple RDs and associated costs, we relied on claims data from three sources: 2019 Medicare Standard Analytical File (SAF, Medicare 5%), 2016 Transformed Medicaid Statistical Information System (TMSIS, Medicaid), and 2018 Optum de-identified Normative Health Information System (dNHI, a large, geographically diverse commercial claims database).

We estimated RD prevalence based on U.S.-specific claims: the Medicare 5% sample for the Medicare population, TMSIS claims for Medicaid population including children covered under the Children's Health Insurance Program (CHIP) and adults 65 or younger, and the dNHI claims for the privately insured population 65 or younger. In each database, we identified patients with 379 different RDs using ICD-10 codes and categorized them into 16 RD groups for adults and 7 RD groups for children (< 18 years), as the sample size was too small to report estimates individually for all 16 RD groups. This allowed us to increase the sample size and to be consistent with the disease groups used in the survey study.

### Direct medical costs

To quantify the annual excess medical costs associated with RD, we compared the average total cost of people with RD with that of a matched comparison group with similar characteristics including age group, gender, race/ethnicity, and insurance type (i.e., commercial, Medicare, or Medicaid), but without any RD. Specifically, we stratified both the patient group and the group without disease based on age, gender, race/ethnicity, and insurance type, we then randomly selected individuals from the group without disease to match with the patient group for each disease, resulting in multiple matches (up to 10) depending on the sample size of a specific stratum. A twelve-months of continuous eligibility in a single year or eligibility until the date of death was required for inclusion in the sample.

Medical costs based on claims data included primary payer paid amount, patient out-of-pocket expenses (e.g., copay, co-insurance, deductibles), and any third party paid amount. We estimated the direct medical costs of RD by types of healthcare service: acute and non-acute inpatient stay; outpatient care; physician office visit; DME; other ancillary, outpatient-based drug administration; retail prescription drug use; and caregiver payments (by Medicaid). Since the Medicare 5% data do not include Part-D claims, we used commercial per-person prescription cost to impute the Medicare per-person prescription cost for each age and disease group. Rx cost for the 60–64-year-olds in the private claims was applied to those Medicare-eligible and 65 and older. All cost estimates were expressed in 2019 dollars. The average direct medical cost was calculated for each of the 16 RD groups for adults and the 7 RD groups for children. We used the group average across all RDs to compare with the comparison group to derive the average RD-attributable cost.

### Indirect costs, non-medical costs, and healthcare costs not covered by insurance

We worked with a broad coalition of patient advocacy organization partners to design an online survey to estimate cost due to reduced labor market participation, productivity loss for those in the labor force, non-medical costs of RD (such as the cost of hiring professional non-medical caregivers to assist with daily living, necessary home modification costs), and disability benefits. The respondents were persons with RD. However, the family member most familiar with the health of the person with RD could respond to the survey, if the health of the person with RD prevented accurate self-reporting, or if the person was a minor.

We took a convenience sample approach and disseminated the survey to the RD communities via partner networks of more than 200 partner-patient advocacy organizations, reaching a broad range of RDs and a large patient sample.

We received 3,484 responses with 1,399 being fully completed. The survey asked about the respondents’ disease and 400+ RDs were reported. After removing misspellings, RD alternative names, and diseases that were not rare (e.g., cancer) or represented a protein, there were 379 unique RDs.

Additional file 3 provides the breakdown of the respondents’ self-description and shows that 57% of the respondents were people with a RD, and 41% of responses were from a family caregiver. About 28% of the responses represent children (< 18 years); the rest were adults, with those above age 65 representing 14% of all responses (Additional file 4**)**. People with RDs were predominantly white (87%), followed by multi-racial individuals (4%). About 69% of the persons with RD attained a high school or above diploma and about 40% attained a Bachelor’s degree or higher. About 77% of people with RD had at least one caregiver (a primary caregiver) and about half had both a primary and a secondary caregiver; 23% of the RD sample did not rely on a caregiver.

Given the survey sample size, the number of RD groups, age stratification, and the number of indirect and non-medical cost components, it was not feasible to calculate reliable cost estimates for all RD groups-age strata. Where the strata sample size permitted, we calculated average costs for that stratum; if the strata sample size had less than 5 observations, we reported the average costs across all RD groups. The mapping of cost components to RD group-age strata is reported in Additional file 5; cost component calculations are detailed below.

To ensure that the early termination of employment was related to RD, we calculated labor market employment-related earnings loss due to RD as the count of persons with RD, who have retired or stopped working in the past 12 months and indicated that RD played a major role in their decision, multiplied with the median annual earnings by job status (full-time versus part-time) obtained from the 2019 American Community Survey public use microdata sample. We used medians rather than averages, as medians are less likely affected by outliers. As the full-time/part-time status of persons with RD before retirement was unknown, we used the allocation of full-time to part-time job status among currently working persons with RD. Then, we calculated earnings loss due to early retirement for those who retired due to RD as a weighted average between those assumed working full-time before retirement and those working part-time before retirement.

We calculated two measures of reduced labor market productivity for those who are employed: absenteeism, (increased workdays missed due to illness), and presenteeism, (illness-related poorer work performance while on the job). We asked about the number of days in an average working month during 2019 the person with RD and the caregivers missed work or felt less productive while at work because of RD. Based on responses by both caregivers and persons with RD, to these two questions and the average daily earnings calculated from the self-reported annual earnings*,* we calculated the productivity loss due to absenteeism by multiplying the number of days missed with the daily earnings and then annualized the total loss. Presenteeism was calculated similarly, with an adjustment factor applied to each day felt unproductive, reflecting that an unproductive day is not equivalent of a total loss of a whole day’s value. The adjustment factor was obtained from the responses to productivity self-assessment scale: i.e., on days when feeling less productive, on average how the productivity of the person with RD and caregivers was affected on a scale from 0 to 10, where 0 represents “not at all”, 1–3 “mildly”, 4–6 “moderately”, 7–9 “markedly”, and 10 represents “extremely”. We translated these responses into the reduced overall productivity (e.g., 0 corresponds to 100% productivity, 10 corresponds to 0% productivity or the full reduction in productivity). Daily earnings were calculated from the annual earnings brackets that were applicable to the respondent in 2019 (categorical responses were converted into numerical values based on the mid-point of each earnings category: everyone who indicated earnings “less than $1,000” was assigned earnings of $500, etc.).

Additionally, RD may affect patients’ and the caregivers’ ability to participate in various social activities using their leisure time [15]. The challenge of quantifying social productivity is measuring the time forgone from social activities and in the proper valuation of the time forgone. Although one could argue that forgone leisure time visiting family and friends also create economic loss, we focused on activities that directly involved volunteering and provide a conservative estimate of the social productivity loss. We asked about the number of hours the person with RD and the caregivers spent in a typical week before and after RD started having a significant impact, on the following social activities: performing voluntary or charity work; providing help to family/friends/neighbors unrelated to personal care or care for person with RD; participating in a political or community-based organization.

We compared self-reported volunteering hours before RD with the average national annual volunteering hours obtained from the Current Population Survey (CPS) Volunteer Supplement that measures the population’s participation in volunteer activities (2017). The national average volunteering hours are generally lower than the self-reported volunteering hours (e.g., 1.9 h per week versus 12.1 h per week for person with RD before RD). Therefore, we took a conservative approach in our calculations by calculating the percentage of people volunteered and average hours volunteered from CPS and multiplied with the estimated percentage productivity loss from the Survey (calculated as the difference between before and after hours divided by before hours) for the three activities combined. Productivity loss due to forgone volunteering activities was calculated as volunteering hours affected per year times $27.20, which is a dollar value per volunteering hour according to the Independent Sector [16].

The non-medical costs calculated included expenses of purchasing formal care (e.g., adult day care and personal aides) and necessary modification to homes, purchases of adapted motor vehicles or car modifications for accessibility, medical foods, dietary supplements, specialty clothing (e.g., compression stockings), and increased transportation costs for medical visits. We also asked about healthcare services not covered by insurance such as experimental treatments, alternative or non-traditional treatments (alternative therapies, massage therapy, acupuncture), and over-the-counter drugs. We estimated non-medical costs and non-covered costs by multiplying the weighted percentage of families who responded as having such expenses and the average expense per-family per-year.

To capture the overall economic burden of RDs, it is always an important policy perspective to be able to identify the extent to which individuals are transitioning into public programs, and what the potential costs to public programs are due to any specific condition/disease, particularly if these costs are avoidable. For example, the Social Security Disability Insurance (SSDI) and the Supplemental Security Income (SSI) are considered as transfer payments (i.e., a cost to one person is a benefit to another person). Therefore, these components may inform on the extent of government budgetary burden due to a specific disease. We asked respondents whether the person with RD had received SSI, SSDI, or other types of disability income, in 2019. While we estimated the average and total disability income due to RD, these costs were excluded from the overall burden estimates, as these funds could have been used for healthcare payments or non-medical expenses already captured in other cost components.

## Supplementary Information

**Additional file 1.** Pre-Person Cost of RD in 2019 by Disease Group and Cost Category (Age < 18). Pre-Person Cost of RD in 2019 by Disease Group and Cost Category (Age ≥ 18). Provides per-person cost by rare disease group and cost category, for each age group.

**Additional file 2.** Mapping of Rare Diseases Into 16 Disease Groups (DG). Provides mapping of rare diseases to corresponding ICD-10 codes and corresponding rare disease group.

**Additional file 3.** Analysis Sample from the Survey. Provides a breakdown of responses received in the online survey by self-description.

**Additional file 4.** Demographic Characteristics and Disease Duration for Persons with RD from the Survey. Provides a breakdown of survey analysis sample by demographic characteristics.

**Additional file 5.** Mapping of Indirect & Non-Medical Cost Components to Disease Group (DG). Provides mapping of cost components to rare disease group.

## Data Availability

The data that support the findings of this study were obtained using standard contracts and data use agreements. The private, Medicare, and Medicaid claims datasets for this study are proprietary to Optum and CMS and, therefore, cannot be shared without a data use agreement with Optum and CMS, respectively. Parties interested in the survey data need to contact the EveryLife Foundation for Rare Diseases for permission to use.

## Abbreviations

CHIP: Children's Health Insurance Program
dNHI: Optum de-identified Normative Health Information System
ICD-10: International Classification of Disease 10th Edition
RD: Rare Disease
SAF: Medicare Standard Analytical File
SSI: Supplemental Security Income
SSDI: Social Security Disability Insurance
TMSIS: Transformed Medicaid Statistical Information System
U.S.: United States

## Footnotes

1 Orphanet is a European website providing information about orphan drugs and rare diseases. It contains content both for physicians and for patients. Its administrative office is in Paris and its official medical journal is the Orphanet Journal of Rare Diseases published on their behalf by BioMed Central. Orphanet codes refer to a unique clinical coding system/nomenclature for rare diseases that aligns with other existing international terminologies and reference databases (including ICD-10, ICD-11, SNOMED-CT, OMIM, UMLS, MeSH, MedDRA, and GARD). Weblink is available at: https://www.orpha.net/consor/cgi-bin/index.php.
