# Supplementary material for: The national economic burden of rare disease in the United States in 2019
Source: Orphanet J Rare Dis. 2022 Apr 12;17:163. doi: 10.1186/s13023-022-02299-5 (PMC9004040; doi:10.1186/s13023-022-02299-5)
Supplement: Supplementary file 1 — Additional file 1. Pre-Person Cost of RD in 2019 by Disease Group and Cost Category (Age < 18). Pre-Person Cost of RD in 2019 by Disease Group and Cost Category (Age ≥ 18). Provides per-person cost by rare disease group and cost category, for each age group. [file 13023_2022_2299_MOESM1_ESM.docx]

**Additional file 1**

**Per-Person Cost of RD in 2019 by Disease Group and Cost Category (Age <18)**

| **Rare Disease Group** | **RD Prevalence (per 10,000)** | **Per-Person Cost** | | | | |
| --- | --- | --- | --- | --- | --- | --- |
|  |  | **Direct Medical Costs** | **Indirect Costs due to Productivity Loss*** | **Non-Medical Costs** | **Healthcare Costs Not Covered by Insurance** | **Total Cost** |
| Chromosomal abnormalities, not elsewhere classified | 3.7 | $40,877 | $30,482 | $17,306 | $2,320 | $90,985 |
| Congenital malformations, deformations and chromosomal abnormalities | 36.4 | $38,483 | $25,712 | $13,499 | $2,396 | $80,090 |
| Diseases of the musculoskeletal system and connective tissue | 7.2 | $21,041 | $32,962 | $15,616 | $2,302 | $71,922 |
| Diseases of the nervous system | 35.4 | $41,880 | $28,430 | $17,078 | $2,306 | $89,694 |
| Lysosomal storage diseases | 0.9 | $132,757 | $34,822 | $18,498 | $2,933 | $189,010 |
| Other endocrine or metabolic disorders | 11.7 | $72,285 | $23,227 | $9,476 | $1,733 | $106,722 |
| Combined Diseases** | 51.8 | $46,750 | $37,791 | $11,185 | $2,062 | $97,788 |
| **Any Rare Disease** | **132.3** | **$32,037** | **$34,448** | **$12,310** | **$1,642** | **$80,437** |

Source: RD prevalence and direct medical costs are calculated from the 2018 Optum de-identified Normative Health Information (dNHI) claims, 2019 Medicare 5% claims, and 2016 Medicaid claims combined with the Census population projection for 2019.

* Per capita indirect cost includes costs for person with RD, and costs to primary caregiver and secondary caregiver of persons with RD.

** Due to small sample sizes among children, rare disease groups such as congenital malformations and deformations of the musculoskeletal system, diseases of the blood and blood-forming organs, diseases of the circulatory system, diseases of the digestive system, diseases of the eye and adnexa, diseases of the respiratory system, diseases of the skin and subcutaneous tissue, immunodeficiency, neoplasms, and other, which are not individually displayed for age group <18, are combined into one group – Combined Diseases.

**Per-Person Cost of RD in 2019 by Disease Group and Cost Category (Age ≥18)**

| **Rare Disease Group** | **RD Prevalence (per 10,000)** | **Per-Person Cost** | | | | |
| --- | --- | --- | --- | --- | --- | --- |
|  |  | **Direct Medical Costs** | **Indirect Costs due to Productivity Loss*** | **Non-Medical Costs** | **Healthcare Costs Not Covered by Insurance** | **Total Cost** |
| Chromosomal abnormalities, not elsewhere classified | 3.8 | $27,497 | $33,017 | $5,200 | $2,122 | $67,837 |
| Congenital malformations and deformations of the musculoskeletal system | 2.8 | $22,219 | $21,981 | $1,821 | $2,875 | $48,896 |
| Congenital malformations, deformations and chromosomal abnormalities | 77.4 | $30,681 | $26,261 | $1,819 | $1,582 | $60,343 |
| Diseases of the blood and blood-forming organs | 80.3 | $54,933 | $14,244 | $2,024 | $848 | $72,049 |
| Diseases of the circulatory system | 287.7 | $46,061 | $19,347 | $4,638 | $2,353 | $72,398 |
| Diseases of the digestive system | 84.4 | $36,762 | $30,346 | $4,972 | $2,076 | $74,156 |
| Diseases of the eye and adnexa | 119 | $13,415 | $24,104 | $1,431 | $1,894 | $40,845 |
| Diseases of the musculoskeletal system and connective tissue | 173.4 | $23,274 | $26,032 | $1,668 | $2,184 | $53,158 |
| Diseases of the nervous system | 326.5 | $31,299 | $27,890 | $6,189 | $2,200 | $67,578 |
| Diseases of the respiratory system | 17.1 | $59,446 | $19,245 | $1,696 | $571 | $80,958 |
| Diseases of the skin and subcutaneous tissue | 70.1 | $25,611 | $16,205 | $1,583 | $2,407 | $45,806 |
| Immunodeficiency | 40.1 | $26,557 | $33,590 | $1,824 | $4,053 | $66,025 |
| Lysosomal storage diseases | 6.8 | $56,087 | $22,493 | $11,812 | $1,673 | $92,065 |
| Neoplasms | 64.3 | $58,025 | $24,082 | $2,031 | $2,467 | $86,605 |
| Other | 51.8 | $20,549 | $34,922 | $2,664 | $3,292 | $61,428 |
| Other endocrine or metabolic disorders | 228.7 | $41,627 | $21,030 | $2,515 | $2,239 | $67,412 |
| **Any Rare Disease** | **1,422.2** | **$28,623** | **$27,501** | **$4,007** | **$2,514** | **$62,644** |

Source: RD prevalence and direct medical costs are calculated from the 2018 Optum de-identified Normative Health Information (dNHI) claims, 2019 Medicare 5% claims, and 2016 Medicaid claims combined with the Census population projection for 2019.

* Per capita indirect cost includes costs for person with RD, and costs to primary caregiver and secondary caregiver of persons with RD.
