# Supplementary material for: The national economic burden of rare disease in the United States in 2019
Source: Orphanet J Rare Dis. 2022 Apr 12;17:163. doi: 10.1186/s13023-022-02299-5 (PMC9004040; doi:10.1186/s13023-022-02299-5)
Supplement: Supplementary file 2 — Additional file 2. Mapping of Rare Diseases Into 16 Disease Groups (DG). Provides mapping of rare diseases to corresponding ICD-10 codes and corresponding rare disease group. [file 13023_2022_2299_MOESM2_ESM.docx]

**Additional file 2**

### **Mapping of Rare Diseases Into 16 Disease Groups (DG)**

| Rare Disease Group (Age group) | Rare Disease | Mapped ICD-10 |
| --- | --- | --- |
| DG11: Chromosomal abnormalities, not elsewhere classified  (<18; 18+) | 15Q11.2 Microdeletion 15Q24 Microdeletion Syndrome 22Q11 Chromosome Duplication Syndrome  22Q13, Phelan-Mcdermid Syndrome 2Q37 Deletion Syndrome 7Q11.23 Duplication Syndrome Angelman Syndrome Chromosome 19 Microdeletion Syndrome Chromosome 21Q Deletion Ddx3X Syndrome Fragile X Syndrome Jacobsen Syndrome Mef2C Deletion Partial Trisomy 16Q Phelan-Mcdermid Syndrome Satb2-Associated Syndrome Syngap1 Syndrome Tetrasomy X Turner Syndrome Williams Syndrome | Q93.5X, Q92.5, Q92.8, Q93.51, Q93.0, Q99.2, Q97.1, Q96.9, Q93.82 |
| DG12: Congenital malformations and deformations of the musculoskeletal system  (18+) | Ehlers Danlos Syndrome Hypermobile Ehlers-Danlos Syndrome Hypochondroplasia Klippel Feil Syndrome Mccune-Albright Syndrome Osteogenesis Imperfecta Polyostotic Fibrous Dysplasia Short Rib Polydactyly Syndrome Type 2 | Q79.60, Q79.62, Q77.4, Q76.1, Q78.1, Q78.0, Q77.2 |
| DG13: Congenital malformations, deformations and chromosomal abnormalities  (<18; 18+) | Aarschot-Scott Syndrome Absence Corpus Callosum Adnp Syndrome Agenesis Of The Corpus Callosum Alagille Syndrome Alport Syndrome Aplasia Cutis Congenita Arnold Chiari Syndrome Arteriovenous Malformation Autosomal Recessive Alport Syndrome Blue Rubber Bleb Nevus Syndrome Cardiofaciocutaneous Syndrome Ccm3 Gene Mutation Chiari Malformation Choanal Atresia Cloves Syndrome Constitutional Mismatch Repair Deficiency (Cmmrd) Dandy Walker Syndrome Dlg4 Epidermolysis Bullosa Floating Harbor Syndrome Foxg1 Syndrome Hereditary Lymphedema Heterotaxy Syndrome Hypohidrotic Ectodermal Dysplasia Hypoplasia Of The Corpus Callosum Hypoplastic Left Heart Syndrome Ichthyosis Isolated Congenital Asplenia Li Fraumeni Syndrome Loeys-Dietz Syndrome Marfan Syndrome Mastocytosis Mayer-Rokitansky-Kuster-Hauser Syndrome Neurofibromatosis Type 1 Noonan Syndrome Pachygyria Peters Anomaly Pfeiffer Syndrome Pppd Prader-Willi Syndrome Primary Ciliary Dyskinesia Primary Congenital Lymphedema Pseudobulbar Affect Rieger Syndrome Shones Complex Slc1A4 Deficiency Snyder-Robinson Syndrome Sticklers Syndrome Sturge-Weber Syndrome Tethered Cord Syndrome Tuberous Sclerosis Complex Unilateral Microphthalmia Vacterl Syndrome Von Hippel Lindau Disease X-Linked Hypohidrotic Ectodermal Dysplasias | Q87.1X, Q04X, Q87.0, Q44.7, Q87.81, Q84.8, Q07.0X, Q28.2, Q87.8X, Q27.8, Q28.3, Q30.0, Q87.3, Z15.09, Q03.1, D43.0, Q81X, F84.8, Q82.0, Q89.3, Q82.4, Q23.4, Q80X, Q89.01, Z15.01, Q87.4, Q82.2, Q52.0, Q85.01, Q87.19, Q04.3, Q13.4, F45.9, Q87.11, Q34.8, F48.2, Q13.8X, Q23.8, Q02, Q89.8, Q85.8, Q06.8, Q85.1, Q11.2, Q87.2 |
| DG14: Diseases of the blood and blood-forming organs  (18+) | Cnot3 Mutation Congenital Dyserythropoietic Anemia Erdheim Chester Disease Hemophilia Idiopathic Thrombocytopenia Purpura Severe Chronic Neutropenia - Autoimmune Sickle Cell Disease Thalassemia Unspecified Severe Chronic Neutropenia Warm Hemolytic Anemia | D68.52, D64.4, D76.3, D68.311, D69.3, D70.8, D57.0X, D57.1X, D57.2X, D56X, D70.9, D59.11 |
| DG15: Diseases of the circulatory system  (18+) | Arterial Fibromuscular Dysplasia Cadasil Catecholaminergic Polymorphic Ventricular Tachycardia Fibromuscular Dysplasia Generalized Lymphatic Anomaly Idiopathic Pulmonary Hypertension Long Qt Syndrome Median Arcuate Ligament Syndrome Moyamoya Non-Genetic Ascending/Descending Aortic Dissection Postural Orthostatic Tachycardia Syndrome Pots Primary Lymphedema Prolong Qt Pulmonary Hypertension | I77.3, I67.850, I47.2, I89.8X, I27.0, I45.81, I77.4, I67.5, I71.01, I49.8, I82.0, I27.20, I27.21 |
| DG16: Diseases of the digestive system  (18+) | Achalasia Amalogenesis Imperfecta Autoimmune Pancreatitis Chronic Intestinal Pseudo-Obstruction  Eosinophilic Esophagitis Lymphocytic Colitis Mesenteric Panniculitis Microvillus Inclusion Disease Primary Biliary Cholangitis Psuedo Obstruction Sclerosing Mesenteritis Short Bowel Syndrome Short Gut | K22.0, K00.5, K86.1, K59.8X, K20.0, K52.832, K65.8, P78.3, K83.01, K65.4, K91.2 |
| DG17: Diseases of the eye and adnexa  (18+) | Acoustic Neuroma Bilateral Vestibular Disorder Bilateral Vestibular Hypofunction Bilateral Vestibular Loss With Oscillopsia Bosch Boonstra Optric Atrophy Syndrome 5Q 15-21 Microdeletions Central Vestibular Disorder Chronic Vestibular Neuritis Duane Syndrome Hyperacusis With Pain Kearns-Sayres Syndrome Leber Congenital Amaurosis Mal De Debarquement Meniere's Disease Osteopetrosis Otosclerosis Semi-Circular Canal Disorder Superior Semicircular Canal Disorder Vestibular Disorder Visual Vestibular Mismatch | D33.3, H81.9X, H47.2X, H81.4, H50.81, H93.23X, H49.81, H35.5X, H81.8X, H81.0X, Q78.2, H80X, H83.8X |
| DG18: Diseases of the musculoskeletal system and connective tissue  (<18; 18+) | Anca Vasculitis Ankylosing Spondylitis Autoimmune Vasculitis Bechet Disease Behcet's Disease Chronic Recurrent Multifocal Osteomyelitis Dermatomyositis Diffuse Systemic Scleroderma Diffused Systematic Sclerosis Fibrodysplasia Ossificans Progressiva  Ghorom-Stout Disease Gorham-Stout Disease Granulomatosis With Polyangiitis Hypocomplementemic Urticarial Vasculitis Hypodermitis Sclerodermiformis Or Lipodermata Sclerosis Juvenile Dermatomyositis Limited Cutaneous Systematic Sclerosis Limited Scleroderma Limited Systemic Sclerosis Micro Angiitis Vasculitis P-Anca Microscopic Polyangiitis Myositis Polymyositis Progressive Familial Intrahepatic Cholestasis Progressive Systemic Sclerosis Sapho Scleroderma Shrinking Lung Disease/Syndrome Sjogrens Syndrome Systemic Diffuse Scleroderma Systemic Juvenile Idiopathic Arthritis Systemic Scleroderma Systemic Sclerosis Takayasu Arteritis Tarlov Cyst Disease Thrombotic Thrombocytopenia Purpura Urticarial Vasculitis Vasculitis | M31X, M45X, M35.2, M86.3X, M33X, M34.9X, M34X, M61.1X, M89.5X, M31.3, M31.8, M33.0X, M31.7, M60X, M60.0, M60.1, M60.2, M60.8X, M33.2X, M32.13, M35.0X, M08.2X, M31.4, M71.38, M31.1 |
| DG19: Diseases of the nervous system  (<18; 18+) | Aicardi Gouteres Syndrome Alper's Syndrome Alternating Hemiplegia Of Childhood Amyotrophic Lateral Sclerosis Arachnoiditis Atp6V1A Gene Mutation Autoimmune Autonomic Ganglionopathy Autoimmune Encephalitis Becker Muscular Dystrophy Cacna1S Cataplexy Cdkl5 Deficiency Disorder Centronuclear Myopathy Charcot-Marie-Tooth Chronic Immune Demyelinating Polyneuropathy Collagen 6 Congenital Muscular Dystrophy Communicating Hydrocephalus Complex Regional Pain Syndrome Congenital Titinopathy Corticobasal Syndrome Also Referred To Corticobasal Degeneration Dravet Syndrome Duchenne Muscular Dystrophy Dysautonomia Dystonia 27 Facioscapulohumeral Muscular Dystrophy Familial Dysautonomia Friedreich Ataxia Giant Axonal Neuropathy Gnao1 Gene Mutation Guillain-Barre Syndrome Hereditary Inclusion Body Myopathy Hereditary Spastic Paraplegia Huntington Disease Hypokalemic Periodic Paralysis Idiopathic Hypersomnia Inclusion Body Myositis Kennedy's Disease Kleine Levin Syndrome Lama Ii Muscular Dystrophy (Merosin Deficient) Lama2 Congenital Muscular Dystrophy Landau-Kleffner Syndrome Lennox-Gastaut Syndrome Limb Girdle Muscular Dystrophy Multifocal Motor Neuropathy Multiple System Atrophy Muscular Dystrophy Myasthenia Gravis Myotonic Muscular Dystrophy Myotubular Myopathy Narcolepsy Nemaline Myopathy Neurodegeneration With Brain Iron Accumulation Neuromyelitis Optica Non 24 Ohtahara Syndrome Olivopontocerebellar Atrophy Primary Hypersomnia Primary Lateral Sclerosis Ryanodine Cardiac Dystrophy Schwartz Jampal Syndrome Scn8A Sepn1 Related Myopathy (Subtype Of Congenital Muscular Dystrophy Small Fiber Polyneuropathy Spinal Cerebellar Atrophy Spinal Muscular Atrophy Spinocerebellar Ataxia Stxbp1 Encephaolopathy With Epilepsy Syringomyelia Titinopathy Transverse Myelitis Trigeminal Neuralgia Trpv4 Tubulinopathy Tuba1A Ullrich Congenital Muscular Dystrophy Vcp Disease West Syndrome X-Linked Myotubular Myopathy | G31.8X, G31.81, G98X, G12.21, G03.9, G40.3X, G99.0, G04.81, G71.01, G72.3, G47.411, G47.421, G40.42, G71.2X, G60.0, G61.81, G91.0, G56.4X, G90.5X, G31.85, G40.83X, G90.1, G24.1, G71.02, G11.11, G60.8, G61.0X, G72.41, G72.9, G11.4, G10, G47.11, G12.1, G47.13, G71.09, G40.80X, G40.81X, G61.82, G90.3, G71.0X, G70.0X, G71.11, G47.41X, G71.21, G23.0, G36.0, G47.2X, G23.8, G47.11, G47.12, G47.13, G47.411, G47.419, G12.23, G71.1,Q78.8, G71.1,Q78.9, G62.89, G62.81, G62.82, G11.8, G12.9, G93.49, G95.0 , G37.3, G50.0, G12.2X, G23.9, G71.20, G40.82X, G71.220 |
| DG20: Diseases of the respiratory system  (18+) | Copa Syndrome Idiopathic Pulmonary Fibrosis Non-Specific Interstitial Pneumonia | J84.8X, J84.112 |
| DG21: Diseases of the skin and subcutaneous tissue  (18+) | Bullous Pemphigoid Dermatitis Herpetiformis Lichen Planopilaris Mucous Membrane Pemphigoid Ocular Cicatricial Pemphigoid Paraneoplastic Pemphigus Pemphigus Vulgaris Pityriasis Rubra Pilaris Prurigo Nodularis Psoriatic Arthritis | L12.0, L13.0, L66.1, L12.1, L10.81, L10.0, L44.0, L28.1, L40.50 |
| DG22: Immunodeficiency  (18+) | 22Q11.2 Deletion Syndrome Burning Mouth Syndrome Cardiac Sarcoidosis Chronic Sarcoidosis Common Variable Immune Deficiency Hereditary Angioedema Mast Cell Activation Syndrome Pulmonary Sarcoidosis Ryr1 Gene Mutation Sarcoidosis Secondary Reynauds Severe Combined Immunodeficiency Disorder Specific Antibody Deficiency | D82.1, T30.0, D86.85, D86X, D83, D84.1, D89.4, D86.0, T88.3X, T69.1X, D81.0, D81.1, D81.2, D81.31, D80.8 |
| DG24: Lysosomal storage diseases  (<18; 18+) | Alexander Disease Adult Onset Batten Disease Canavan Disease Ddost-Cdg Fabry Gaucher's Gm1 Gangliosidosis Hunter Syndrome Hurler Syndrome Late Onset Tay-Sachs Lysosomal Storage Diseases Mucolipidosis Mucopolysaccharidosis Type 1 Mucopolysaccharidosis Type 2 Mucopolysaccharidosis Type 4A Multiple Sulfatase Deficiency Niemann-Pick Type C Pompe Pseudo Hurler Polydystrophy Sanfilippo Syndrome Undiagnosed Leukodystrophy | E75.2X, E75.4, E75.29, E77.8, E75.21, E75.22, E75.19, E76.02, E76.01, E75.02, E75.5, E75.11, E77.0, E77.1, E76.0X, E76.1, E76.210, E76.211, E75.26, E75.242, E74.02, E77.0, E76.22 |
| DG25: Neoplasms  (18+) | Chronic Myelogenous Leukemia With P230 Breakpoint Cns Lymphoma Cutaneous T-Cell Lymphoma Desmoid Tumor Desmoid Type Aggressive Fibromatosis Kaposiform Lymphangiomatosis Leiomyosarcoma Lymphangiomatosis Mastocytosis Mesenchymal Chondrosarcoma Metastatic Leiomyosarcoma Multiple Myeloma Neuroendocrine Tumors Paraganglioma Pheochromocytoma Polycythemia Vera Recurrent Respiratory Papillomatosis Sacral Chordoma Synovial Sarcoma Thymoma Waldenstroms Macroglobulinemia | C92.1X, C83.84, C84.AX, D48.1, D18.1, C49.9, D47.0X, D47.09, C41.9, C90.0X, C7A, D44.7, C74.10, C74.11, C74.12, D45, D14.4, C41.2, D15.0, C37, C88.0 |
| DG31: Other  (18+) | Cyclic Vomiting Syndrome Diaphragmatic Endometriosis Endosalpingiosis  Focal Segmental Glomerulosclerosis (FSGS) Minimal Change Disease Mycobacterium Avium Complex Nontuberculous Mycobacteria Nontuberculous Mycobacterial Infection Ramsey Hunt Syndrome | R11.15, N80.8, N94.89, N04.0, A31.0, A31.9, B02.21 |
| DG32: Other endocrine or metabolic disorders  (<18; 18+) | Acromegaly Addisons Disease Adiposis Dolorosa Adrenal Insufficiency Adrenoleukodystrophy Adult Polyglucosan Body Disease Alpha-1 Antitrypsin Deficiency Amyloidosis Ataxia Barth Syndrome Congenital Adrenal Hyperplasia Creatine Transporter Deficiency Cushing's Disease Cystic Fibrosis Cystinuria Familial Chylomicronemia Syndrome Familial Hypercholesterolemia Familial Partial Lipodystrophy Fcs Familial Chylomicronemia Syndrome Glycogen Storage Disease Hermansky Pudlak Syndrome Homozygous Familial Hypercholesterolemia Hyperinsulinism Hyperammonemia Syndrome Hypoparathyroidism Hypophosphatemic Ricketts Lchad Deficiency Lipodystrophy Lipomatosis Dolorosa Type 2 Lipoprotein Lipase Deficiency Methylmalonic Acidemia Mitochondrial Disease Mitochondrial Metabolism Disorder Neonatal Onset Multisystem Inflammatory Disease  Organic Acidemias Peroxisomal Biogenesis Disorder Phenylketonuria Porphyria Primary Adrenal Insufficiency Primary Hyperoxaluria Type 1 Pten Hamartoma Tumor Syndrome Tyrosinemia Urea Cycle Disorder Wilson Disease Wolfram-Like Syndrome X-Linked Hypophosphatemia Zellwegers Spectrum Disorder | E22.0, E27.1, E88.2, E27.40, E71.521, E74.09, E88.01, E85X, R27.0 , E78.71, E25.0, E72.9, E24, E83.39X, E72.01, E78.3, E78.01, E88.1, E74.00, E74.01, E74.03, E74.04, E74.09, E70.331, E72.8X, E21.0, E21.1, E21.2, E83.39, E71.310, E71.111, E88.4X, E85.0X, E71.11X, E71.510, E70.0, E80.0, E80.1, E80.1X, E74.810, E71.440, E70.21, E72.2X, E83.01, E13.8, E83.31 |
