# Supplementary material for: The national economic burden of rare disease in the United States in 2019
Source: Orphanet J Rare Dis. 2022 Apr 12;17:163. doi: 10.1186/s13023-022-02299-5 (PMC9004040; doi:10.1186/s13023-022-02299-5)
Supplement: Supplementary file 3 — Additional file 3. Analysis Sample from the Survey. Provides a breakdown of responses received in the online survey by self-description. [file 13023_2022_2299_MOESM3_ESM.docx]

**Additional file 3**

**Analysis Sample from the Survey**

| Which of the following best describes you (the person who is responding to the survey)? | Frequency | Percent | Cumulative |
| --- | --- | --- | --- |
| A person living with a rare disease | 802 | 56.9 | 56.9 |
| A family caregiver for someone who has a rare disease | 572 | 40.6 | 97.5 |
| A paid caregiver for someone who has a rare disease | 1 | 0.1 | 97.6 |
| A family member of someone who has a rare disease, but not a direct caregiver (e.g., family member who is not responsible for organizing/providing day-to-day care) | 21 | 1.5 | 99.1 |
| A close friend to someone who has a rare disease, but not a caregiver | 3 | 0.2 | 99.3 |
| Sub-total | 1,399 | 99.3 | 99.3 |
| Do not have a rare disease and do not know anyone with a rare disease | 10 | 0.7 | 100.0 |
| Total number of respondents | 1,409 | 100 | 100 |

Source: Primary data collected through the survey.
