# Supplementary material for: The national economic burden of rare disease in the United States in 2019
Source: Orphanet J Rare Dis. 2022 Apr 12;17:163. doi: 10.1186/s13023-022-02299-5 (PMC9004040; doi:10.1186/s13023-022-02299-5)
Supplement: Supplementary file 4 — Additional file 4. Demographic Characteristics and Disease Duration for Persons with RD from the Survey. Provides a breakdown of survey analysis sample by demographic characteristics. [file 13023_2022_2299_MOESM4_ESM.docx]

**Additional file 4**

**Demographic Characteristics and Disease Duration for** **Persons with RD from the Survey**

|  | Age | |
| --- | --- | --- |
|  | **Number** | **Percent** |
| < 18 | 379 | 27.8 |
| ≥ 18 | 984 | 72.2 |
| Total | **1,360** | **100** |
|  | **Race/Ethnicity** | |
|  | **Number** | **Percent** |
| American Indian or Alaska Native | 11 | 0.8 |
| Asian | 22 | 1.6 |
| Black or African American | 33 | 2.4 |
| Multi-racial | 50 | 3.7 |
| Native Hawaiian or Other Pacific Islander | 1 | 0.1 |
| White or Caucasian | 1,188 | 87.4 |
| Other | 23 | 1.7 |
| Prefer not to answer | 32 | 2.4 |
| Total | **1,360** | **100** |
|  | **Education Level** | |
|  | **Number** | **Percent** |
| Less than a high school diploma | 224 | 16.5 |
| High school diploma (General Education Diploma or equivalent) | 110 | 8.1 |
| Some College (1-4 years, no degree) | 180 | 13.2 |
| Associate degree (AS, AAS, etc.) | 103 | 7.6 |
| Bachelor’s degree (BA, BS, etc.) | 265 | 19.5 |
| Master’s degree (MA, MS, etc.) | 185 | 13.6 |
| PhD or Professional School Degree (MD, JD, etc.) | 88 | 6.5 |
| Not applicable | 202 | 14.9 |
| Prefer not to answer | 2 | 0.1 |
| Do not know | 1 | 0.1 |
| Total | **1,360** | **100** |
|  | **Duration** | |
|  | **Since the 1st Symptom** | **Since Diagnosis** |
| Less than 5 years (% of responses) | 19.5 | 37.7 |
| 5-9 years (in %) | 20.1 | 23.7 |
| 10-14 years (in %) | 18.4 | 15.7 |
| 15 -19 years (in %) | 10.7 | 7.8 |
| 20 years or more (in %) | 31.3 | 15.1 |
| Total | **100** | **100** |
| Mean (in years) | 16.5 | 10.2 |

Source: Primary data collected through the Survey.
