# Supplementary material for: The national economic burden of rare disease in the United States in 2019
Source: Orphanet J Rare Dis. 2022 Apr 12;17:163. doi: 10.1186/s13023-022-02299-5 (PMC9004040; doi:10.1186/s13023-022-02299-5)
Supplement: Supplementary file 5 — Additional file 5. Mapping of Indirect & Non-Medical Cost Components to Disease Group (DG). Provides mapping of cost components to rare disease group. [file 13023_2022_2299_MOESM5_ESM.docx]

**Additional file 5**

**Mapping of Cost Components, including Indirect Cost, Non-Medical Cost, and Healthcare Costs Not Covered by Insurance, to Disease Group (DG)**

|  | Person with RD | |
| --- | --- | --- |
| Cost component | **<18 (7 disease groups)** | **≥18 (16 disease groups)** |
| Loss due to forced retirement | NA | Aggregate 16gr |
|  |  | (N=91) |
| Absenteeism | NA | **16gr** |
|  |  | (N=394) |
| Presenteeism | NA | **16gr** |
|  |  | (N=394) |
| Reduced social productivity | **7gr** | **16gr** |
|  | (N=45) | (N=721) |
| Healthcare costs not covered by insurance | 7gr | 16gr |
|  | (N=359) | (N=952) |
| Healthcare facility/institutions use | 7gr | 16gr |
|  | (N=362) | (N=917) |
| Formal non-med care | **7gr** | **Aggregate 16gr** |
|  | (N=114) | (N=136) |
| Necessary home modification | **7gr** | **16gr** |
|  | (N=106) | (N=171) |
| Special equipment at home or on a personal family vehicle | 7gr | **16gr** |
|  | (N=214) | (N=498) |
| Transportation costs | 7gr | 16gr |
|  | (N=318) | (N=690) |
| Other costs: home schooling | 7gr | NA |
|  | (N=82) |  |
| Missed schooling | 7gr*** | NA |
|  | (N=278) |  |
| Special education | 7gr*** | NA |
|  | (N=311) |  |
| Fin assistance from charitable org | **Aggregate 7gr** | **Aggregate 16gr** |
|  | (N=27) | (N=53) |
| SSI | **Aggregate 7gr** | **Aggregate 16gr** |
|  | (N=34) | (N=152) |
| SSDI | Aggregate 7gr | **16gr** |
|  | (N=6) | (N=182) |
| OTDI | **Aggregate 7gr** | **Aggregate 16gr** |
|  | (N=24) | (N=136) |

Note: SSDI - Social Security Disability, SSI - Supplemental Security Income, OTDI - other types of disability income.

|  | Primary Caregiver | |
| --- | --- | --- |
| Cost component | **<18 (7 disease groups)** | **≥18 (16 disease groups)** |
| Loss due to forced retirement | Aggregate 7gr | Aggregate 16gr |
|  | (N=4) | (N=35) |
| Absenteeism | 7gr | **16gr** |
|  | (N=225) | (N=439) |
| Presenteeism | 7gr | **16gr** |
|  | (N=225) | (N=439) |
| Reduced social productivity | 7gr | 16gr |
|  | (N=287) | (N=534) |

|  | Secondary Caregiver | |
| --- | --- | --- |
| Cost component | **<18 (7 disease groups)** | **≥18 (16 disease groups)** |
| Loss due to forced retirement | Aggregate 7gr | Aggregate 16gr |
|  | (N=9) | (N=7) |
| Absenteeism | 7gr | **16gr** |
|  | (N=273) | (N=205) |
| Presenteeism | 7gr | **16gr** |
|  | (N=273) | (N=205) |
| Reduced social productivity | 7gr | **16gr** |
|  | (N=214) | (N=239) |

Note: Seven DG groups for children include DG11; DG13; DG18; DG19; DG24; DG32; Other (which includes all other 10 DGs). 16 DG groups for adults are: DG11; DG12; DG13; DG14; DG15; DG16; DG17; DG18; DG19; DG20; DG21; DG22; DG24; DG25; DG31; DG32. List of DGs with the corresponding RDs is listed in Table A-1. Abbreviations: 7gr and 16gr indicate that DG-specific estimates are calculated for each of 7 (16) groups. **7gr** and **16gr** indicate that for the majority of DGs, the DG-specific estimates are calculated for each group, but for some DGs, due to small sample sizes, aggregate estimates across 7 (16) groups are calculated. “Aggregate 7gr” and “Aggregate 16gr” indicate that sample sizes did not permit reporting of any DG-specific estimate and only aggregate is calculated; “**Aggregate 7gr**” and “**Aggregate 16gr**” indicate that for the majority of DGs the aggregated estimate is calculated, but for a few DGs, where sample size permitted, DG-specific estimates are calculated. * For missed schooling and special education, we used survey data to calculate percent of population who had these services or missed school for each of 7 DGs; per person dollar values are from the external sources.
